# The complete mitochondrial genome of common redshank (*Tringa totanus*)

**DOI:** 10.1080/23802359.2019.1629350

**Published:** 2019-07-12

**Authors:** Jianying Ren, Xue Jiang, Liang Dou, Lin Yan, Xuelian Zhang, Xiuyue Zhang

**Affiliations:** a Key Laboratory of Bio-Resources and Eco-Environment (Ministry of Education), College of Life Sciences, Sichuan University, Chengdu, China;; b Sichuan University Museum, Sichuan University, Chengdu, China;; c Sichuan Key Laboratory of Conservation Biology on Endangered Wildlife, College of Life Sciences, Sichuan University, Chengdu, China

**Keywords:** Common redshank, *Tringa tetanus*, complete mitochondrial genome

## Abstract

*Tringa totanus* is declining throughout Europe and performing protection in China. In this study, we sequenced the complete mitochondrial genome using PCR method. It is the first published complete mitochondrial genome of *T. tetanus*, which is a circular molecule of 16,818 bp, and it contains 13 typical vertebrate protein-coding genes, 22 tRNA, and two rRNA. The phylogenetic tree was constructed to validate the taxonomic status of *T. totanus*, exhibiting it a closer relationship to *T. glareola*.

The common redshank (*Tringa totanus*) is a wader species with a wide breeding distribution throughout the Palearctic. The taxonomy is rather complicated and at least six subspecies have been recognized (Ottvall et al. 2005). Mitochondrial DNA (mtDNA) has been widely used in molecular ecology, phylogeography study, and many intractable phylogenies (Robert et al. 2011). Attention in species identification falls on mammalian species primarily and on occasion insect species but rarely do avian species. In addition, it is difficult to distinguish shorebirds because of few opportunities for close observation, complex molting patterns and light factors for colour perception (Thompson et al. 1990). Mitochondrial DNA genes have been used to identify closely related avian species (Boonseub et al. 2009). But so far, only seldom and partial sequences about *T. totanus* are deposited at NCBI.

In this study, we determined the complete mitochondrial genome of *T. totanus* from an individual collected at Aba Hongyuan Airport, Sichuan Province, China. The sample was kept in the Key Laboratory of Bioresources and Ecoenvironment, Sichuan University. The total DNA was extracted from the muscle tissue. The tRNA predicted using tRNAscan-SE-2.0, and other genes are identified by homology alignment with other mitogenome of *Tringa* avian.

The circular mitochondrial genome of *T. tetanus* is 16,818 bp in length. The overall composition of the base is 31.8% A, 25.2% T, 29.6% C, and 13.4% G, with a total G + C ratio of 43%. The genome contains 13 typical vertebrate protein-coding genes (PCGs), 22 transfer RNA (tRNA) genes, two ribosome RNA (rRNA) genes, and one control region, which is the same as other avian in *Tringa* genus. ND3 gene has a frameshift phenomenon at *T. ochropus*, but not in *T. tetanus* (Chen et al. 2016). The annotated mitogenome of *T. totanus* has been deposited in GenBank under accession No. MK922124.

The start codon of 13 protein-coding genes is ATG except for *COI* start with GTG. TAN is the most frequent stop codon, and AGN and T–– are also occurred. The 12S rRNA and 16S rRNA genes are 969 bp and 1592 bp, respectively. Among all genes, *tRNA-Gln*, *tRNA-Ala*, *tRNA-Tyr*, *tRNA-Asn*, *tRNA-Cys*, one *tRNA-Ser*, *tRNA-Pro*, *ND6*, and *tRNA-Glu* are encoded on the L-strand and others are encoded on the H-strand.

In order to understand the relationship of *Tringa* avian (whole mitochondrial sequence accessible), we constructed the phylogenetic tree (maximum-likelihood) based on the concatenated DNA sequence of 13 mitochondrial PCGs of *Tringa semipalmata* (NC 036016.1), *Tringa erythropus* (NC 030585.1), *Tringa glareola* (NC 039096.1), *Tringa ochropus* (NC 033974.1), *Tringa tetanus*, and *Scolopax rusticola* (NC 025521.1) (Figure 1). The result showed that *T. totanus* has closer relationship with *T. glareola*. This study improves our understanding of the evolution relationship of mitochondrial DNA in *Tringa.*

**Figure 1.**
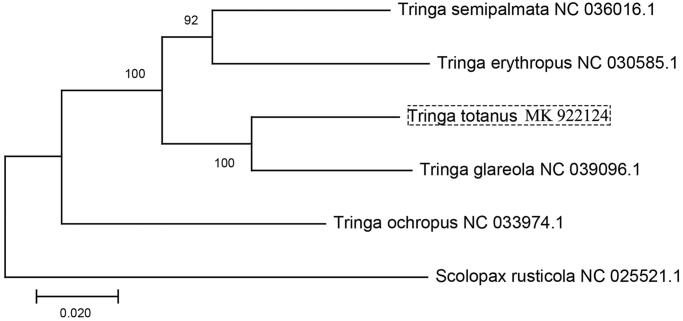
Phylogenetic tree of maximum likelihood (ML) method based on the mitochondrial PCGs nucleotide sequences of published *Tringa* species. Numbers represent node supports inferred from bootstrap support values.
